# Cure for Squint without Operation

**Published:** 1883-07-20

**Authors:** 


**Cure of Squint Without Operation.**—In the early stages of a convergent strabismus, before the internal rectus muscle is permanently contracted, Dr. Boucheron (Schmidt's Jarbucher, January 17, 1883) claims that a cure is possible without operation. He states that, as a convergence is caused by efforts of accommodation for near objects, if we take away the power of accommodation, squint will not occur. He maintains a constant mydriasis by the instillation of atropine night and morning. A cure is usually obtained in two or three weeks. If atropine is not well borne, other mydriatics, such is duboisia, may be used. In nine cases of intermittent strabismus the author obtained eight cures by this method.—*Medical Record, N. Y.*

